# Brugada Syndrome: More than a Monogenic Channelopathy

**DOI:** 10.3390/biomedicines11082297

**Published:** 2023-08-18

**Authors:** Antonella Liantonio, Matteo Bertini, Antonietta Mele, Cristina Balla, Giorgia Dinoi, Rita Selvatici, Marco Mele, Annamaria De Luca, Francesca Gualandi, Paola Imbrici

**Affiliations:** 1 Department of Pharmacy-Drug Sciences, University of Bari “Aldo Moro”, 70125 Bari, Italy; antonella.liantonio@uniba.it (A.L.); antonietta.mele@uniba.it (A.M.); giorgia.dinoi@uniba.it (G.D.); marco.mele@uniba.it (M.M.); annamaria.deluca@uniba.it (A.D.L.); 2 Cardiological Center, Sant’Anna University Hospital of Ferrara, 44121 Ferrara, Italy; doc.matber@gmail.com (M.B.); bllcst@unife.it (C.B.); 3 Medical Genetics Unit, Department of Mother and Child, Sant’Anna University Hospital of Ferrara, 44121 Ferrara, Italy; rita.selvatici@unife.it; 4 Cardiothoracic Department, Policlinico Riuniti Foggia, 71122 Foggia, Italy

**Keywords:** Brugada syndrome, *SCN5A*, Nav1.5, cardiac channelopathy, arrhythmia

## Abstract

Brugada syndrome (BrS) is an inherited cardiac channelopathy first diagnosed in 1992 but still considered a challenging disease in terms of diagnosis, arrhythmia risk prediction, pathophysiology and management. Despite about 20% of individuals carrying pathogenic variants in the *SCN5A* gene, the identification of a polygenic origin for BrS and the potential role of common genetic variants provide the basis for applying polygenic risk scores for individual risk prediction. The pathophysiological mechanisms are still unclear, and the initial thinking of this syndrome as a primary electrical disease is evolving towards a partly structural disease. This review focuses on the main scientific advancements in the identification of biomarkers for diagnosis, risk stratification, pathophysiology and therapy of BrS. A comprehensive model that integrates clinical and genetic factors, comorbidities, age and gender, and perhaps environmental influences may provide the opportunity to enhance patients’ quality of life and improve the therapeutic approach.

## 1. Clinical Diagnosis and Risk Stratification

Brugada syndrome (BrS) is an inherited cardiac channelopathy, first diagnosed in 1992. It is electrophysiologically characterized by a typical type 1 ECG pattern displaying a coved ST-segment elevation of at least 2 mm followed by a negative T wave in at least one right precordial lead and by a high incidence of life-threatening arrhythmic events in the absence of overt structural heart disease [1]. BrS could be responsible for 4–12% of all sudden cardiac death (SCD) and for up to 20% of SCD that results from polymorphic ventricular tachyarrhythmias (VT) or ventricular fibrillation (VF) [2]. Clinically, BrS presents with syncope or cardiac arrest due to VF, and the ECG pattern indicates abnormal electrical activity in the upper part of the right ventricle outflow tract (RVOT). The ECG type 1 pattern may be observed either spontaneously or after being unmasked by a provocative drug test with a sodium-channel blocker, such as ajmaline, flecainide, pilsicainide or procainamide [3]. These antiarrhythmic drugs, by inhibiting inward sodium current (INa), increase the imbalance between inward and outward currents in early phases of the action potential (AP), thus revealing the phenotypic expression of the BrS [2]. However, most of the patients are asymptomatic. 

The incidence and prevalence of BrS varies around the world but is estimated to be up to 10 in every 10,000 in Europe/USA, with the highest prevalence (up to 94 in every 10,000) in Southeast Asia [4]. The mean age of patients with BrS is 41 ± 15 years; nevertheless, arrhythmic events have been reported from the age of 2 up to 84 [5]. BrS occurs predominantly in males (up to 90%), suggesting that hormones are involved in BrS pathophysiology, especially in adults [6,7]. Arrhythmias typically occur while sleeping (nocturnal agonal breathing), at rest, or following large meals; this suggests that a high vagal tone may increase arrhythmic risk. Furthermore, fever is an important risk factor for ECG changes that may enhance conduction slowing in the RVOT and subsequently lead to VF [8,9,10]. Among the disease comorbidities, gastrointestinal complications and ischemic heart disease have been reported [5,11].

**Figure 1:**
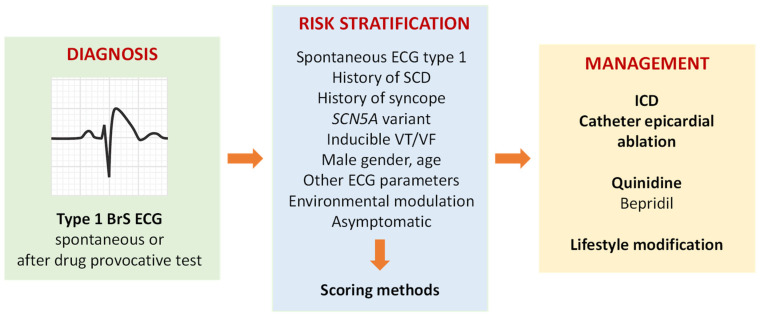
Management of BrS patients.

Unlike other inherited arrhythmia syndromes, the first manifestation of the disease in patients with BrS can be SCD [12]. Therefore, the identification of appropriate biomarkers for accurate risk stratification in patients with BrS is fundamental for their management. Some risk factors for BrS have been defined by current clinical guidelines (2022) and are universally accepted, including symptoms, spontaneous type 1 ECG parameters, age, sex, clinical history and family history of SCD and the presence of *SCN5A* rare variant [12,13]. Patients at the highest risk are easily identified because they may present to the clinic after an aborted SCD or manifest very characteristic clinical and ECG features. Conversely, the management of asymptomatic patients and those at low risk (drug-induced patients) has not yet been defined. Risk stratification to support therapeutic decision-making also involves aging patients with BrS, as they may develop comorbidities such as ischemic heart disease [5]. Apparently, retrospective studies evaluating differences between younger and elderly patients with BrS suggest that patients with BrS who were >60 years of age, and certainly for those >70 years of age, have a better prognosis than those <60 years of age [5] over a long follow-up period. Given the limited predictive power of single parameters, risk-scoring models that incorporate multiple predictive factors (genetic, electrophysiological and environmental factors, age and sex) have been proposed and are required to improve risk stratification in BrS patients [14,15]. The Shangai Score System is an example [16]. Thus, BrS may be a multifactorial disorder, wherein multiple genetic and environmental factors each contribute to varying extents (Figure 1). 

## 2. Management 

According to current guidelines, patients who are survivors of an aborted SCD or have documented spontaneous sustained VT or with spontaneous type 1 ECG and syncope are candidates for an implantable cardioverter defibrillator (ICD) [3]. However, as said before, most patients with spontaneous type 1 are asymptomatic and with an expectedly low annual rate of arrhythmic events. So, an ICD indication in these patients needs careful consideration. Studies in elderly BrS patients provided a solid basis to defer prophylactic ICD therapy in patients with BrS who are >60 years of age and certainly in those >70 years of age [5]. However, alternative treatment options are not readily available. 

Quinidine, a drug that blocks the transient outward potassium current (Ito) (and thus able to reduce the ionic imbalance in phase 1 of the AP), has been shown to be beneficial in patients in which ICD is contraindicated, with multiple ICD shocks, arrhythmic storms or in children at high risk [2,17,18]. In patients with a history of appropriate VF-suppressing ICD shocks, chronic oral treatment with denopamine, cilostazol (phosphodiesterase type 3 inhibitor and platelet antiaggregant), and bepridil (calcium channel blocker) is efficacious in terminating VF [17]. In patients with electrical storms, isoproterenol is effective in VF suppression [17]. 

In more recent years, RVOT epicardial ablation appeared to successfully reduce the incidence of arrhythmias and eliminate the ECG pattern in BrS patients and is recommended in patients with recurrent appropriate ICD shocks refractory to drug therapy. Clearly, it is too early to perform epicardial ablation in asymptomatic patients [2,8]. 

Several drugs that block the cardiac sodium channel Nav1.5 need to be avoided, including psychotropic drugs and anesthetics ([19]; www.brugadadrugs.org accessed on 28 July 2023). Specific lifestyle adjustments are appropriate for all BrS patients, with fever and alcohol intake, avoided as well [20]. 

## 3. Genetics and Molecular Mechanisms

### 3.1. Oligogenic Disease with Incomplete Penetrance and Expressivity 

As SCD might be the first presenting symptom of BrS, early diagnosis becomes essential. In up to 35% of the BrS cases, a causal variant can be identified (positive genotype). Yet, most of the affected individuals (approximately 65%) remain genetically undetermined (negative genotype), and for this reason, identifying new susceptibility genes for BrS is necessary. This represents a big gap in knowledge of the genetics of this disease [2]. 

To date, variants in >25 different genes have been linked to BrS, including genes encoding ion channel subunits and regulatory proteins [20]. According to the American College of Medical Genetics and Genomics (ACMG), gene variants can be classified into five categories: pathogenic, likely pathogenic, uncertain significance, likely benign, and benign by in silico prediction tools [21]. Although the distinction between pathogenic and benign variants is crucial for the therapeutic management of genotype-positive BrS patients and to prevent SCD, the interpretation of genetic results is difficult. In this framework, appropriate functional in vitro assays addressing the effects of a variant on channel activity and expression represent a powerful tool for testing pathogenicity and risk stratification as well as for investigating variant-specific pharmacological approaches. Variants have been found in *SCN5A*, *SCN10A*, *SCN1B-3B*, *GPD1L*, *RANGRF*, *SLMAP*, *ABCC9*, *KCNH2*, *KCNE3*, *KCNJ8*, *KCNE5*, *KCND3*, *HCN4*, *CACNA1C*, *CACNB2B*, *CACNA2D1*, *TRPM4*, and *PKP2*, among others. Many of these identified variants have been found in single families and are only responsible for less than 5% of the BrS cases; thus, their association with BrS is still questionable [12,20]. 

The *SCN5A* gene, encoding for the alpha subunit of the cardiac voltage-gated sodium channel Nav1.5, accounts for ~20–30% of BrS cases and it is the only one considered clinically valid. Among >300 different variants in *SCN5A* identified (http://www.ncbi.nlm.nih.gov/clinvar accessed on 28 July 2023), 31% are frameshift, nonsense or splice-site variants, and 69% are missense or rarely in-frame deletions/insertions [22]. Variants in the *SCN5A* gene are linked to various clinical phenotypes, including LQTS3 (gain-of-function), isolated cardiac conduction disease (loss-of-function) and sinus node dysfunction (loss-of-function) [12,23,24]. In addition, genetic variants in *SCN5A* have also been associated with atrial fibrillation and heart failure susceptibility [25] and with dilated cardiomyopathy [26]. Therefore, it is not surprising that several *SCN5A* variants manifest clinically as overlapping syndromes (i.e., combinations of BrS, LQTS, conduction disorders, sick sinus syndrome, and structural defects). This clinical overlap could be explained by the assumption that pathogenic variants can alter various properties of the Nav1.5 channel, such as gating, interaction with auxiliary subunits, and gene expression, to varying degrees [20]. In some cases, a single *SCN5A* variant can lead to different arrhythmic phenotypic traits in the same family or even in single patients [27,28,29,30]. Moreover, in some pedigrees, some affected family members lack the familial *SCN5A* variant, suggesting other origins for the disease [28,31]. 

Altogether, the incomplete penetrance and variable expressivity of BrS suggest that the disease most probably involves the combined contribution of different genes and variants of variable impact. For instance, the presence of copy number variations (CNVs) in genes affecting the onset of BrS is emerging as a possible mechanism in BrS [32]. In addition, the number of common variants identified in BrS patients is increasing (for example, single nucleotide polymorphisms in *SCN10A*, *SCN5A*, *HEY2,* and *ZFHX3* genes), even though their possible association with BrS is still unclear [29,32]. Some of these variants might act as genetic modifiers that, alongside the primary defect, may exacerbate the severity of the disease or may protect a carrier of a primary genetic defect from developing the disease, thus explaining the interindividual variability in disease expression [29,32]. Furthermore, a large proportion of detected variants, such as those affecting RNA splicing or outside protein-coding regions, are classified by in silico prediction tools as variants of uncertain significance (VUS), and unless a functional assay is performed, their clinical relevance in BrS pathogenesis remains questionable [29,32]. Although BrS was initially described as a monogenic autosomal dominant disease with incomplete penetrance (Mendelian disease), there is now increasing evidence that it may follow a more complex genetic model. Indeed, it may be considered an oligogenic or polygenic disease in which more than one causal gene contributes to producing a clinical phenotype (the ‘multiple genetic hit hypothesis’) [9,32]. This assumption is not surprising when one considers that the QRS complex in a human ECG is regulated by several genes, some of which are already involved in BrS and other cardiac disorders.

The influence of genetic testing on the risk of cardiac arrhythmia and prognosis is still debated [33,34]. It remains unclear to what extent different gene variants increase the risk of arrhythmic events or SCD, and so they alone may not be effective in risk stratification. However, genetic data may provide a complementary tool for risk stratification, and familial screening is recommended once a genotype-positive BrS patient has been diagnosed. Importantly, the occurrence of a causative *SCN5A* variant(s), while not necessarily leading to active treatment, should lead to lifestyle modifications (i.e., avoidance of medications, fever and high-dose alcohol consumption). 

To better understand the complex genetic architecture of BrS and assess the impact of rare and common variants on risk prediction, whole exome sequencing (WES) studies have been recently performed. One large-scale genome-wide association meta-analysis study of unrelated BrS cases showed a high polygenic risk score supporting the contribution of cumulative common risk alleles among different patient subgroups, as well as genetic associations with cardiac electrical traits and disorders in the general population [35]. The study also highlighted that transcriptional regulation of sodium channels is a key feature of BrS pathogenesis and susceptibility, as indicated by the predominance of cardiac transcription factor loci in the association analysis. Another study performed in eight BrS families carrying an *SCN5A* variant did not show any clear genetic modifier nor risk factors for SCD; nevertheless, this study highlighted the possible role of the cholesterol pathway, the development of fibrosis, as well as the circadian rhythm, as modulators of BrS phenotype [36].

### 3.2. SCN5A Variants: Genotype–Phenotype Correlation

Two principal molecular mechanisms have been assumed to explain the BrS phenotype: either a decrease in INa currents or an increase in Ito currents during early repolarization [2]. Cardiac INa currents are mainly carried by the activity of the voltage-gated sodium channel Nav1.5 which is expressed predominantly in cardiomyocytes. This fast inward current principally sustains the rapid depolarization that accounts for the AP upstroke and is fundamental to initiating the multi-step excitation–contraction coupling cascade in cardiomyocytes. Under physiological conditions, Nav1.5 activation and inactivation processes are strictly voltage regulated to ensure rhythmic cardiac electrical activity. 

**Figure 2:**
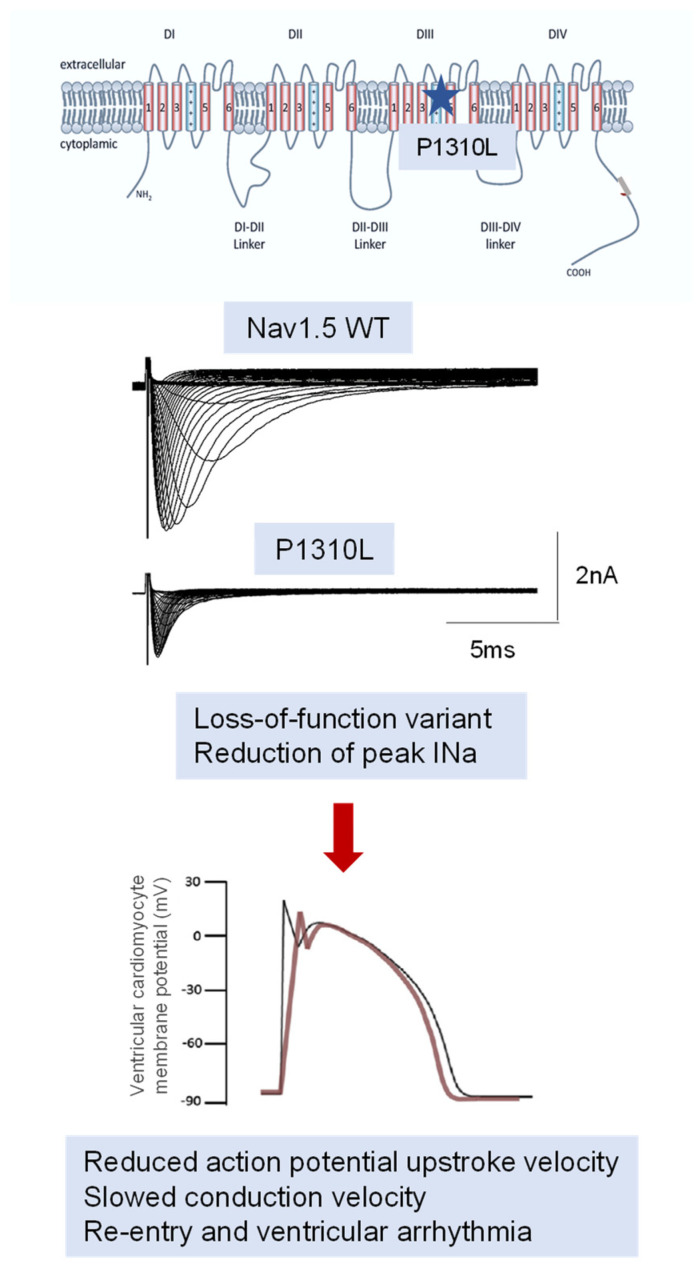
Functional characterization of the Nav1.5 P1310L variant in S4 DIII identified in a family affected by BrS described in [29]. The blue star indicates the location of the P1310L variant on channel structure.

Most *SCN5A* variants associated with BrS are missense variants located in the four transmembrane domains of the channel protein (DI, DII, DIII, or DIV; for instance, E1784K, D356N, G1408R, P1310L). When heterologously expressed, the BrS-bearing mutant Nav1.5 channels show different degrees of loss-of-function by patch clamp recordings of INa. The variants can either impair membrane trafficking or modify channel gating properties or kinetics or cause haploinsufficiency determined by premature transcriptional stop signals. In particular, a decrease in current density, a positive shift in the activation curve, a negative shift in the inactivation curve, or a loss of regulation by intracellular factors such as PKA, ankyrin G or a1-syntrophin have been described [22,29,37,38,39,40,41] (Figure 2). 

Consistently, cardiomyocytes derived from mice models or patients’ stem cells displayed a reduction in INa density and a reduction in AP upstroke velocity and altered ECG parameters [42]. 

Increasing evidence also supports the role of *SCN5A* CNVs as a cause of BrS and encourages the screening of *SCN5A* CNVs in BrS patients [32]. So far, a few CNVs, duplications and deletions potentially associated with the disease have been described in this gene [32]. Four CNVs, identified in probands who were symptomatic or had a family history, were described as producing a non-functional or severely damaged Nav1.5 protein. The clinical characteristics of the probands carrying these CNVs were severe and similar to those of patients carrying truncation or missense variants causing >90% of peak INa reduction [43]. CNVs in the *SCN5A* gene can provide a possible new mechanism for BrS, and further studies are needed to clarify their clinical significance in BrS pathogenesis.

As mentioned in the previous paragraph, the influence of *SCN5A* variants on risk assessment has been largely explored. Different studies suggest that cohorts of BrS patients carrying *SCN5A* variants are associated with more severe phenotypes with respect to patients without *SCN5A* variants [44,45,46,47]. Moreover, at least two studies show that different *SCN5A* variants may have a different impact on INa, highlighting the role of functional assays in assessing the risk for asymptomatic family members. In particular, the location (pore, S5, P loop, S6) and the type (missense vs. truncation) of *SCN5A* variants may be related to the clinical severity of the disease. For instance, missense variants, which most often disrupt the gating of the channel with less disruptive effects on the cardiac sodium channel, are associated with less severe phenotypes; in contrast, truncation variants, producing truncated protein usually not inserted into the sarcolemma, cause haploinsufficiency (greater INa decrease) and are associated with a more severe phenotype and a poorer prognosis, including higher incidence of syncope, increase in PR and QRS interval post drug challenge [48]. Several case studies have reported that *SCN5A* pore variants cause a stronger reduction in the sodium current in heterologous expression systems [49,50]. In another study, the presence of a pore *SCN5A* variant was associated with higher values of P-wave, PQ interval and QRS duration and higher risk of cardiac events in 7 years follow-up in a large cohort [46]. Most *SCN5A* missense loss-of-function variants exert a dominant negative effect. This class of variant confers a particularly high risk of BrS [51]. Conversely, a single study on 1029 patients (Finger registry) observed that the presence of an *SCN5A* variant was not predictive of arrhythmic events [52]. 

Some *SCN5A* polymorphisms have been identified in BrS patients. For example, the common variant H558R seems to be a genetic modulator of BrS among carriers of a pathogenic *SCN5A* variant, mitigating the clinical phenotype [29]. In addition, Nav1.5 dysfunction may affect other ion channel proteins and accessory subunits at channelosomes in the cardiac muscle cell and vice versa [2,53,54,55,56]. For instance, Nav1.5 and Kv4.3 channels modulate each other’s function via trafficking and gating mechanisms [57]. Deletion in the transient receptor potential melastatin member 4 (TRPM4) encoding a Ca^2+^-activated, non-selective cation channel expressed in atrial and ventricular cardiac myocytes unexpectedly reduces the peak INa currents in murine cardiac myocytes [58]. Trafficking deficiency and retention of the Kir2.1 channel at the Golgi apparatus affect the translocation of Nav1.5 channels at the plasma membrane [59]. This modulation may have important implications for the pathogenesis and treatment of cardiac syndromes. Furthermore, there is now increasing evidence that functional changes in Nav1.5 may affect both ionic and non-ionic events that, alone or in combination, contribute to arrhythmogenesis [53,60,61] (see below).

### 3.3. Variants in Genes Encoding for Other Ion Channels and Regulatory Proteins

Variants in genes encoding three Nav1.5 beta-subunits (*SCN1B*, *SCN2B* and *SCN3B*) have been described in BrS patients. These disrupt Nav1.5 trafficking and reduce INa in cell lines [2,62,63,64]. 

Recent data show that the Nav1.8 channel (*SCN10A* gene) is a modulator of cardiac conduction, and *SCN10A* variants have been associated with atrial fibrillation (AF) and BrS [65,66,67,68]. *SCN10A* variants seem to influence the duration of the PR and QRS interval, heart rate (HR) and the risk of arrhythmias [65,66]. However, as the expression level (extremely low) and function of the Nav1.8 in the heart are still controversial, the real contribution of *SCN10A* as a susceptibility gene for BrS has not been determined yet [2,69]. One possibility is that *SCN10A* variants may alter the *SCN5A* gene expression level and act as a disease modifier [65,66,70].

The increase in the transient outward potassium current (Ito) and the decrease in the L-type Ca^2+^ current (ICaL) are also thought to contribute to the pathogenesis of BrS, although only in rare cases. Reduced ICaL, as a consequence of loss-of-function variants of Cav1.2 or the regulatory subunits β2 and α2δ1 (encoded by *CACNA1C*, *CACNB2b* and *CACNA2D1*), respectively, have also been described as a cause of BrS (BrS3) [71]. Interestingly, because ICaL also contributes to the AP plateau duration, loss-of-function variants in these genes have also been associated with a shortening of AP duration and a short QT interval in the ECG, resulting in a blended phenotype of BrS and SQTS [71]. 

Variants in several subunits that affect directly or indirectly the Ito current, responsible for the phase-1 repolarization of the AP, may cause BrS. Gain-of-function variants in *KCND3* and *KCND2* encoding for Kv4.3 and Kv4.2 voltage-dependent potassium channel mediators of the transient Ito current induce BrS by direct increase in Ito currents [57,72]. Sporadic gain-of-function variants in genes coding for Kv4.3 accessory subunits (*KCNE3, KCNE5, KCNAB2*) increase Ito indirectly and also lead to BrS [73,74,75]. By a similar mechanism, variants that induce an increase in other repolarizing currents (IK-ATP and IKr) may predispose to BrS [41,67].

Less is known about the involvement of variants in other ion channels in BrS. One loss-of-function variant in the *HCN4* gene that mediates the If current of the sinoatrial node has been reported in a patient with suspected BrS [76]. Variants in the *TRPM4* channel have also been found in BrS patients. This channel, like *HCN4*, appears to be involved in the diastolic depolarization that leads to the AP in the sinoatrial node [55,77]. Interestingly, both gain-of-function and loss-of-function *TRPM4* variants have been reported in BrS patients, indicating that further studies are required to clarify the role of this channel in the disease [58]. Interestingly, a double heterozygosity for pathogenic variants in *SCN5A* and *TRPM4* has been found in a BrS patient whose parents were heterozygous for each variation [54].

Among the genes that have been sporadically associated with BrS, some encode for regulatory non-ion channel proteins that are directly or indirectly bound to the Nav1.5 channel and can modulate expression, traffic and function and thus INa (Table 1). Altered trafficking and reduced INa is the mechanism also reported for sporadic variants in a series of genes such as *FGF12* (fibroblast growth homologous factor 12), *GPD1L* (glycerol-3-phosphate dehydrogenase 1-like), *SLMAP* (sarcolemma associated protein), *RANGRF* (MOG1), *PKP2* (plakophilin-2) and *MAPRE2* (microtubule plus-end binding protein EB2) [35,78,79,80,81,82,83,84]. 

Despite CNVs being uncommon in BrS, recently, one copy number deletion of *GSTM3* (Glutathione S-Transferase Mu 3) has been identified in Taiwanese BrS patients without SCN5A mutations, associated with reduced INa and higher rates of syncope, suggesting a novel potential genetic modifier/risk predictor for the development of BrS [85]. 

Further, a reduction in *SCN5A* transcription can increase arrhythmia risk and cause BrS. Two missense variants in the transcription factor gene *ZFHX3* encoding the zinc finger homeobox 3 (Zfhx3) have been identified in patients with BrS questioning the role of this gene in BrS pathogenesis and suggesting a potential contribution as a disease modifier [86]. Recently, a rare missense variant was found (G145R) in the cardiac transcription factor *TBX5* (T-box transcription factor 5) in a family negative for the *SCN5A* variant. Electrophysiological recordings from patients-derived cardiomyocytes show a direct *SCN5A* down-regulation, decreased peak INa and enhanced “late” cardiac INa, which were entirely corrected by editing G145R to wild-type [87].

Two regulatory proteins associated with BrS appeared to modulate Ito currents. Loss-of-function variants of *SEMA3A*, which encodes semaphorin-3A, a protein interacting with Kv4.3, lead to increased Ito currents [88]. A recent genome-wide association study found a strong association between a region near the *HEY2* gene and BrS [68]. Although no BrS-associated variant has been identified to date, a genome-wide co-expression study suggested that *KCNIP2*, the beta subunit of the Kv4.2-mediated Ito current, is among the genes regulated by *HEY2* and possibly associated with BrS [2,89]. 

Regarding sporadic variants in minor genes, given the limited number of carriers, the underlying mechanisms or the genotype–phenotype correlation cannot be clearly determined [24]. In addition, several variants in minor BrS genes also occur with relatively high frequency in the general population. Thus, their causative role in the disease is under debate and, as said, to date, *SCN5A* is the only gene considered clinically actionable and disease-causing. Nevertheless, the “mutation load” occurring in BrS strongly suggests the adoption of a gene panel to obtain an accurate genetic diagnosis, which is mandatory for risk stratification, prevention, and therapy.

All the genes so far associated with BrS are listed in Table 1. For the sake of clarity, the other diseases in which these genes are also involved are mentioned.

## 4. Pathophysiological Mechanisms

### 4.1. Preclinical Models

Insight into the pathophysiological mechanisms of BrS represents an important challenge aimed at improving diagnosis and treatment. Routinely, in the presence of a novel variant, in silico studies are used to predict the likelihood of pathogenicity. However, these tools often result in variants of uncertain significance. Thus, family segregation analysis and functional studies are necessary to complement the genetic diagnosis of a single patient. In this scenario, several experimental cell and animal models are currently available, each with peculiar advantages and disadvantages and suitable for studying different aspects of BrS [90].

A relatively easy model to study the functional effect of BrS-associated variants consists of the transfection of mutant genes into heterologous systems such as human embryonic kidney (HEK) cells, SV40 transformed (tsA201) cells or Chinese hamster ovary (CHO) cells. These models allow us to study the impact of single variants on the electrophysiological properties of ion channels in a rapid and cost-effective way and have been used to functionally characterize several *SCN5A* variants, as detailed before [29,39]. Although the heterologous expression of mutant channels does not allow us to perform AP measurements or evaluate conduction velocity, this approach represents the first step to understanding genotype–phenotype correlation.

More comprehensive studies are based on animal models. The murine model is certainly the most used, easy to handle and studied with non-invasive imaging techniques giving readouts that can be translated in clinical settings [91] and are relatively cheap. Homozygous knockout mice for the cardiac sodium channel gene (*Scn5a*^−/−^) are embryonically lethal, likely due to heart structural defects [92]. Most studies have been carried out on the heterozygous knockout mouse (*Scn5a^+/^*^−^*)* and the transgenic model carrying the human variant 1798insD in the *Scn5a* gene (*Scn5a^1798insD/+^*). Compared with the wild type, ventricular cardiomyocytes from *Scn5a^+/^*^−^ mice are characterized by a reduction in *Scn5a* mRNA and protein levels in parallel with a 50% reduction of sodium conductance and an impaired conduction velocity. Specifically, the maximum conduction velocity was higher at the endocardial surface of the right ventricles than at the epicardial surface. Interestingly, studies in this model revealed the greater involvement of the right ventricles compared to the left ones in BrS pathophysiology and suggest that this difference may be related to structural differences consisting of a higher number of non-vascular regions in the right ventricles [93]. In heterozygous mice (*Scn5a^+/^*^−^), cardiac fibrosis develops with age, and the incidence and severity of conduction abnormalities and ventricular arrhythmias correlate with the degree of structural abnormalities that develop in the myocardium [94,95,96]. These features point to an overlap between pathophysiological processes related to BrS and progressive cardiac conduction defects in *Scn5a^+/^*^−^ hearts and suggest that similar molecular and cellular processes may also develop in BrS patients. Electrophysiological studies on the heterozygous *Scn5a^1798insD/+^* mice showed a significant prolongation of PQ, QRS and QTc intervals. Cardiomyocytes derived from these mice displayed a reduction in INa density and prolongation of AP in parallel with a reduction in AP upstroke velocity. All the effects observed in murine animal models correlate with BrS symptoms. However, with respect to humans, murine models are characterized by a different ECG profile, which makes it difficult to correlate strictly with the ECG changes seen in BrS patients [90].

Pigs, dogs and rabbits are less used animal models. Despite these models showing electrophysiological properties and ion channel expression profiles more similar to humans, they are difficult to handle and have high costs.

Today, human-induced pluripotent stem cell–derived cardiomyocytes (hiPSC-CM) represent a patient-specific system that allows us to investigate the expression and the function of genes and proteins at different stages of cell development. Different protocols have been used to direct pluripotent cells into atrial-like or ventricular-like cardiomyocytes. Furthermore, hiPSC-CM represents a precision method to study patient-specific variants that allows correlating the electrophysiological defect to the specific clinical phenotype [32]. These cells may also shed light on the variability between patients carrying the same variant. To date, several hiPSC-CM models have been generated. To study sodium channel properties, iPSC-CM were initially generated from murine models, and the results obtained paved the way for this experimental approach. Subsequently, two iPSC-CM lines from BrS patients carrying two *SCN5A* variants have been produced. These showed a reduction in INa and maximal upstroke velocity of the AP parallel to an increase in triggered activity and impaired calcium handling [42,97]. Similarly, El-Battrawy et al. generated hiPSC-CM from patients carrying two variants, one in *SCN1B* gene (c.629T>C and c.637C>A) and the other in *SCN10A* gene (c.3803G>A and c.3749G>A), that showed a significant reduction in peak and late INa together with a reduction in maximal upstroke velocity of the AP [98,99]. Although hiPSC-CM is a good model to study single-cell electrophysiology in BrS and similar arrhythmias, they have not been used to measure conduction defects recorded instead in other models, such as murine and porcine.

As recommended by ACMG, proper functional assays are required to classify suspected VUS and correctly interpret their protein impact and clinical relevance. Recently, both the minigene assay and hiPSC-CM have been used to reclassify some *SCN5A* VUS splice-altering variants observed in patients with BrS according to each variant’s characteristics. Interestingly, some of these variants have been reclassified as likely pathogenic or pathogenic, supporting the role of rigorous functional studies in resolving uncertainty [100].

### 4.2. Pure Channelopathy or Concealed Cardiomyopathy: The Growing Role of SCN5A

Despite the availability of different disease models, the pathophysiological mechanisms underlying BrS remain to be clarified. The spectrum of severity of the ECG and clinical manifestations of BrS correlate with the existence of various underlying genetic and cellular abnormalities of the right ventricular outflow tract (RVOT) and surrounding structures. One could speculate that BrS is a heterogeneous disease with a common clinical phenotype (ECG abnormalities) potentially explained by more than one pathophysiological mechanism [2,101].

Two principal hypotheses have been proposed to explain the BrS clinical phenotype: the repolarization hypothesis and the depolarization hypothesis. According to the repolarization hypothesis, the reduction in INa may generate an outward shift in the balance of currents in the right ventricular epicardium (transmural dispersion of repolarization between epicardium and endocardium at RVOT) which, if large enough, can lead to circling of electrical impulses around a point as a re-entrant circuit, causing an arrhythmia [12]. Experimental studies have indeed demonstrated that the typical ECG pattern results from an imbalance between inward and outward currents in the early phases of repolarization (phase 1 of the AP), arising either from a reduction in inward currents (mainly INa and less importantly ICaL) or from an increase in outward currents (Ito, IKs, or IKr), as mentioned previously [2]. This imbalance may elicit the development of ventricular arrhythmias through a mechanism of phase 2 re-entry, triggered when the voltage in phase 1 reaches approximately −30 mV. This event may lead to premature heterogeneous repolarization affecting only a subset of cells. This heterogeneous repolarization throughout the cardiac tissue may then promote re-entrant arrhythmias [2].

Alternatively, the depolarization hypothesis claims that the loss of INa, by impairing phase 0 of the AP, likely slows the electrical conduction in the RVOT, thus being primarily responsible for conduction delay and AP re-entry leading consequently to abnormal heart rhythm [101]. Evidence-based studies sustained this latter pathogenetic mechanism over time.

It had been initially thought that BrS was an exclusively electrical disorder (channelopathy) not accompanied by any structural changes [1]. However, although the majority of BrS patients have a structurally normal heart, deep phenotyping has shown the presence of subtle structural alterations in both ventricles of BrS patients [102,103]. Novel approaches to evaluate the arrhythmogenic substrate include ECG imaging to map with high-resolution epicardial electrical activity and MRI to visualize minor structural alterations in the RVOT [103]. These approaches revealed that BrS patients with loss-of-function *SCN5A* variants may show RV hypertrophy, fibrosis, epicardial fatty infiltration, and myocyte cytoplasm degeneration [20,104,105,106]. In addition, genetic and immunohistochemical analyses of autoptic specimens from BrS family members revealed alterations at the tissue and molecular levels. In particular, an increase in epicardial collagen, fibrosis and fatty infiltration, a reduction in connexin43 expression and altered gap junction communication, particularly in the RVOT, were detected [12,89,107]. These alterations are typically associated with cardiomyopathies, and an overlap in clinical as well as molecular features between BrS, arrhythmogenic cardiomyopathy (ACM/ARVC), hypertrophic cardiomyopathy, and laminopathies are emerging [108,109,110]. As in dilated cardiomyopathies, RVOT dilation reduced RV ejection fraction and RV wall motion impairment, in addition to age-related fibrosclerotic degeneration in the RV myocardium, which can also take place in BrS over decades [103,111]. Recently, a longitudinal cardiac MRI study showed that structural changes may evolve over time, with the development of focal fibrosis in a significant proportion of patients with BrS [112]. Regardless of their origin, structural abnormalities can induce conduction block and promote re-entrant arrhythmias. Catheter ablation of the surviving myocardium between fibrotic tissue can eliminate this arrhythmogenic substrate, thus restoring the Brugada ECG pattern and reducing arrhythmic risk in symptomatic patients with recurrent VF events [101,107].

Despite these reports and the association shown in some genetic studies, the role of structural abnormalities (fibrosis), in addition to the primary biophysical changes in BrS, is a matter of ongoing scientific investigation.

## 5. Conclusions

In summary, although BrS is considered a genetic disease with a common clinical phenotype, the molecular mechanism remains unknown in 70–85% of clinically confirmed cases. This is because many patients follow a non-Mendelian inheritance and present rare variants in non-coding regions or unknown genes, or even CNVs [85]. Thus, it is highly likely that disease manifestation, variability and severity can result from a number and interplay of rare and common variants occurring at the genotype level [32] and from more than one pathophysiological mechanism [2,101,113,114]. Despite most BrS cases resulting from variants in *SCN5A*, the emerging picture is that the molecular mechanisms underlying BrS may be far more complex than expected by the sole electrophysiological Nav1.5 defect [115]. The subtle structural abnormalities observed in animal models and in BrS patients may be the consequence of myocardium remodeling, perhaps secondary to altered excitation–contraction coupling and appearing later in life [60,106,116]. Structural abnormalities could explain the occurrence of BrS symptoms in mid-life, suggesting that the underlying disease takes time to develop. Instead, electrical alterations may be responsible for the early-onset symptoms in young patients with BrS, as suggested by the prevalence of *SCN5A* variants in this population [117]. These still unclear molecular and cellular processes may develop with different features in different patients, including those that do not carry *SCN5A* variants. Improved understanding of the heterogeneous pathogenesis of BrS, combining data from various biological sources, will advance the prediction of arrhythmia/SCD risk and provide the basis for a mechanism-based therapy.

## Figures and Tables

**Table 1:** Genes associated with BrS and other diseases.

Class of Genes/Proteins	Gene	Protein	Functional Defect	BrS and Other Related Diseases	References
**Sodium channels and accessory subunits**	*SCN5A*	Sodium channel alpha subunit Nav1.5	Loss-of-function variants reduce Nav1.5 expression and alter gating properties or kinetics, causing reduced INa	BrS 1, Sudden Infant Death Syndrome and LQTS 3	[2]
	*SCN10A*	Sodium channel alpha subunit Nav1.8	Involvement in BrS controversial due to low expression in the heart; may modulate *SCN5A* gene expression level?	BrS, Familial Episodic Pain Syndrome 2 and Sodium Channelopathy-Related Small Fiber Neuropathy	[65,66,70]
	*SCN1B*	Sodium channel beta 1 subunit	Loss-of-function variants cause reduced INa	BrS 5, Familial AF 13, LQTS, SCD and DEE 52	[64]
	*SCN3B*	Sodium channel beta 3 subunit	Loss-of-function variants cause reduced INa	BrS 7, Familial AF, LQTS and SCD	[64]
**Potassium channels and accessory subunits**	*KCND3*	Voltage-Gated Potassium Channel Kv4.3	Gain-of-function variants increase I_to_	BrS 9 and Spinocerebellar Ataxia 19 and 22	[57]
	*KCNE3*	Voltage-Gated Potassium Channel Regulatory Subunit MiRP2	Gain-of-function variants increase I_to_ mediated by Kv4.3	BrS 6 and Hypokalemic Periodic Paralysis Type 1	[73]
	*KCNE5*	Cardiac Voltage-Gated Potassium Channel Regulatory Beta Subunit 5	Gain-of-function variants increase I_to_ mediated by Kv4.3	BrS and Amme Complex	[75]
	*KCNAB2*	Voltage-Gated Potassium Channel Regulatory Beta Subunit 2	Gain-of-function variants increase I_to_ mediated by Kv4.3	BrS, Chromosome 1P36 Deletion Syndrome and Partial Trisomy Distal 4Q	[74]
	*KCND2*	Voltage-Gated Potassium Channel Kv4.2	Gain-of-function variants increase I_to_ mediated by Kv4.2	BrS, LQTS and Early Myoclonic Encephalopathy	[72]
	*KCNJ8*	Inwardly Rectifying Potassium Channel Kir6.1	Gain-of-function variants increase the I_K-ATP_	BrS, Cantu Syndrome and Infant SD	[67]
	*ABCC9*	ATP Binding Cassette Subfamily C Member 9 SUR2	Gain-of-function variants increase the I_K-ATP_ mediated by Kir6.1	BrS, Cantu Syndrome and Familial AF 12	[67]
	*KCNH2*	Voltage-Gated Potassium Channel Kv11.1 (HERG)	Gain-of-function variants increase I_Kr_	BrS, LQTS 2 and SQTS 1	[41]
**Calcium channels and accessory subunits**	*CACNA1C*	Voltage-Gated Calcium Channel Subunit Alpha Cav1.2	Loss-of-function variants reduce I_CaL_	BrS 3, Timothy Syndrome and LQTS 8	[71]
	*CACNB2*	Voltage-Gated Calcium Channel Beta 2 Subunit	Loss-of-function variants reduce I_CaL_	BrS 4 and Lambert-Eaton Myasthenic Syndrome	[71]
	*CACNA2D1*	Voltage-Gated Calcium Channel Auxiliary Subunit Alpha2delta 1	Loss-of-function variants reduce I_CaL_	BrS, Familial SQTS and DEE 110	[71]
**Other ion channels**	*TRPM4*	Transient Receptor Potential Cation Channel Subfamily M Member 4 contributes to depolarization that gives rise to the AP in the SAN	Both gain-of-function and loss-of-function variants cause BrS with unclear mechanisms	BrS, Progressive Familial Heart Block Type Ib and Erythrokeratodermia Variabilis Et Progressiva 6	[54,58,77]
	*HCN4*	Hyperpolarization Activated Cyclic Nucleotide Gated Potassium Channel 4,	Loss-of-function variant reduces I_f_ in the SAN	BrS 8 and SSS 2	[76]
**Non-ion channel proteins that affect Nav1.5 traffick and I_Na_**	*GPD1L*	Glycerol-3-Phosphate Dehydrogenase 1 Like	Variants cause trafficking defects of Nav1.5 and reduction in I_Na_	BrS 2	[80]
	*RANGRF*	RAN Guanine Nucleotide Release Factor (MOG1) (chaperone that binds to Nav1.5 and facilitates Nav1.5 trafficking to the cell surface)	Variants cause trafficking defects of Nav1.5 and likely reduce I_Na_	BrS and SSS	[82,83]
	*SLMAP*	Sarcolemma Associated Protein (Golgi)	Variants cause trafficking defects of Nav1.5 and reduction in I_Na_	BrS and lung cancer	[81]
	*PKP2*	Plakophilin 2	Variants reduce the number of Nav1.5 channels at the intercalated disc and likely reduce I_Na_	BrS, Familial Arrhythmogenic Right Ventricular Dysplasia 9 and ARVC	[84]
	*GPD1L*	Glycerol-3-Phosphate Dehydrogenase 1 Like	Variants cause trafficking defects of Nav1.5 and reduction in I_Na_	BrS 2	[80]
	*FGF12B*	Fibroblast Growth Factor FGF-12b (potent regulator of Nav1.5 traffic and function)	Variants reduce I_Na_ but not I_CaL_	BrS, DEE 47 and Non-Specific Early-Onset Epileptic Encephalopathy	[78,79]
	*MAPRE2*	Microtubule-Associated Protein RP/EB Family Member 2	Variants cause microtubule-related trafficking effects on Nav1.5 expression	BrS, Skin Creases, Congenital Symmetric Circumferential 2 and Multiple Benign Circumferential Skin Creases On Limbs	[35]
	*GSTM3*	Glutathione S-Transferase Mu 3	Copy number deletions cause reduction in I_Na_ and higher rates of syncope and SCD	BrS, Larynx Cancer and Pharynx Cancer	[85]
**Transcription factors that regulate *SCN5A* transcription and I_Na_**	*ZFHX3*	zinc finger homeobox 3	Variants downregulate *SCN5A* transcription and Nav1.5 expression and can modify BrS phenotype	Genetic modifier in BrS, Prostate Cancer and Small Cell Cancer Of The Lung	[86]
	*TBX5*	T-box transcription factor 5	Variants downregulate *SCN5A* transcription, decrease cardiac peak I_Na_ and enhance “late” I_Na_	BrS, Holt-Oram Syndrome and Patent Foramen Ovale	[87]
**Non-ion channel proteins that affect I_to_**	*SEMA3A*	semaphorin-3A binds to Kv4.3 and reduces peak current densities without perturbing cell surface expression	Loss-of-function variants increase I_to_ mediated by Kv4.3	BrS, Hypogonadotropic Hypogonadism 16 with or without Anosmia	[88]
	*HEY2*	Hes Related Family BHLH Transcription Factor With YRPW Motif 2 affects cardiac ion channel gene expression in mice and humans	SNP increasing *HEY2* transcript increases *KCNIP2* expression and I_to_	BrS, Aortic Aneurysm, Familial Thoracic 1 and Tricuspid Atresia	[68,89]

DEE—developmental and Epileptic Encephalopathy; LQTS—long QT syndrome; SQTS—short QT syndrome; AF—Atrial Fibrillation; SCD—sudden cardiac death; ARVC—Arrhythmogenic Right Ventricular Cardiomyopathy; SSS—Sick Sinus Syndrome; SAN—sinoatrial node; SNP—single nucleotide polymorphism.

## Data Availability

Data sharing not applicable.
